# Supplementary material for: Resolving the full spectrum of human genome variation using Linked-Reads
Source: Genome Res. 2019 Apr;29(4):635–45. doi: 10.1101/gr.234443.118 (PMC6442396; doi:10.1101/gr.234443.118)
Supplement: Supplemental Material [file supp_gr.234443.118_Supplemental_longranger-2.2.zip › longranger-2.2.tar.bz2!/lr2.2-public/lib/stan/cmdstan/stan/doc/api/html/mcmc__writer_8hpp_source.html]

Stan: src/stan/io/mcmc\_writer.hpp Source File


|  |  |
| --- | --- |
|  | Stan  2.5.0  probability, sampling & optimization |


- Main Page
- Related Pages
- Namespaces
- Classes
- Files

- File List
- File Members

All Classes Namespaces Files Functions Variables Typedefs Enumerations Enumerator Friends Macros Pages

- src
- stan
- io

mcmc\_writer.hpp

Go to the documentation of this file.

1 #ifndef STAN\_\_IO\_\_MCMC\_\_WRITER\_\_HPP

2 #define STAN\_\_IO\_\_MCMC\_\_WRITER\_\_HPP

3

4 #include <ostream>

5 #include <iomanip>

6 #include <string>

7 #include <vector>

8

9 #include <stan/mcmc/base\_mcmc.hpp>

10 #include <stan/mcmc/sample.hpp>

11 #include <stan/model/prob\_grad.hpp>

12

13 #include <stan/common/recorder/csv.hpp>

14

15 namespace stan {

16

17  namespace io {

18

26  template <class M,

27  class SampleRecorder, class DiagnosticRecorder,

28  class MessageRecorder>

29  class mcmc\_writer {

30

31  private:

32  SampleRecorder& sample\_recorder\_;

33  DiagnosticRecorder& diagnostic\_recorder\_;

34  MessageRecorder& message\_recorder\_;

35

36  std::ostream\* msg\_stream\_;

37

38  public:

39

51  mcmc\_writer(SampleRecorder& sample\_recorder,

52  DiagnosticRecorder& diagnostic\_recorder,

53  MessageRecorder& message\_recorder,

54  std::ostream\* msg\_stream = 0)

55  : sample\_recorder\_(sample\_recorder),

56  diagnostic\_recorder\_(diagnostic\_recorder),

57  message\_recorder\_(message\_recorder),

58  msg\_stream\_(msg\_stream) {

59  }

60

79  void write\_sample\_names(stan::mcmc::sample& sample,

80  stan::mcmc::base\_mcmc\* sampler,

81  M& model) {

82  std::vector<std::string> names;

83

84  sample.get\_sample\_param\_names(names);

85  sampler->get\_sampler\_param\_names(names);

86  model.constrained\_param\_names(names, true, true);

87

88  sample\_recorder\_(names);

89  }

90

91

106  template <class RNG>

107  void write\_sample\_params(RNG& rng,

108  stan::mcmc::sample& sample,

109  stan::mcmc::base\_mcmc& sampler,

110  M& model) {

111  std::vector<double> values;

112

113  sample.get\_sample\_params(values);

114  sampler.get\_sampler\_params(values);

115

116  Eigen::VectorXd model\_values;

117

118  model.write\_array(rng,

119  const\_cast<Eigen::VectorXd&>(sample.cont\_params()),

120  model\_values,

121  true, true,

122  msg\_stream\_);

123

124  for (int i = 0; i < model\_values.size(); ++i)

125  values.push\_back(model\_values(i));

126

127  sample\_recorder\_(values);

128  }

129

138  template <class Recorder>

139  void write\_adapt\_finish(stan::mcmc::base\_mcmc\* sampler,

140  Recorder& recorder) {

141  if (!recorder.is\_recording())

142  return;

143  std::stringstream stream;

144  sampler->write\_sampler\_state(&stream);

145

146  recorder("Adaptation terminated\n" + stream.str());

147  }

148

149

157  void write\_adapt\_finish(stan::mcmc::base\_mcmc\* sampler) {

158  write\_adapt\_finish(sampler, sample\_recorder\_);

159  write\_adapt\_finish(sampler, diagnostic\_recorder\_);

160  }

161

162

175  void write\_diagnostic\_names(stan::mcmc::sample sample,

176  stan::mcmc::base\_mcmc\* sampler,

177  M& model) {

178  std::vector<std::string> names;

179

180  sample.get\_sample\_param\_names(names);

181  sampler->get\_sampler\_param\_names(names);

182

183  std::vector<std::string> model\_names;

184  model.unconstrained\_param\_names(model\_names, false, false);

185

186  sampler->get\_sampler\_diagnostic\_names(model\_names, names);

187

188  diagnostic\_recorder\_(names);

189  }

190

203  void write\_diagnostic\_params(stan::mcmc::sample& sample,

204  stan::mcmc::base\_mcmc\* sampler) {

205  std::vector<double> values;

206

207  sample.get\_sample\_params(values);

208  sampler->get\_sampler\_params(values);

209  sampler->get\_sampler\_diagnostics(values);

210

211  diagnostic\_recorder\_(values);

212  }

213

214

230  template <class Recorder>

231  void write\_timing(double warmDeltaT, double sampleDeltaT,

232  Recorder& recorder) {

233  if (!recorder.is\_recording())

234  return;

235

236  std::string title(" Elapsed Time: ");

237  std::stringstream ss;

238

239

240  recorder();

241

242  ss.str("");

243  ss << title << warmDeltaT << " seconds (Warm-up)";

244  recorder(ss.str());

245

246  ss.str("");

247  ss << std::string(title.size(), ' ') << sampleDeltaT

248  << " seconds (Sampling)";

249  recorder(ss.str());

250

251  ss.str("");

252  ss << std::string(title.size(), ' ')

253  << warmDeltaT + sampleDeltaT

254  << " seconds (Total)";

255  recorder(ss.str());

256

257  recorder();

258  }

259

260

267  void write\_timing(double warmDeltaT, double sampleDeltaT) {

268  write\_timing(warmDeltaT, sampleDeltaT, sample\_recorder\_);

269  write\_timing(warmDeltaT, sampleDeltaT, diagnostic\_recorder\_);

270  write\_timing(warmDeltaT, sampleDeltaT, message\_recorder\_);

271  }

272

273  };

274

275  } //io

276

277 } // stan

278

279 #endif

stan::io::mcmc\_writer::mcmc\_writer

mcmc\_writer(SampleRecorder &sample\_recorder, DiagnosticRecorder &diagnostic\_recorder, MessageRecorder &message\_recorder, std::ostream \*msg\_stream=0)

Constructor.

**Definition:** mcmc\_writer.hpp:51

stan::io::mcmc\_writer::write\_sample\_params

void write\_sample\_params(RNG &rng, stan::mcmc::sample &sample, stan::mcmc::base\_mcmc &sampler, M &model)

Outputs samples.

**Definition:** mcmc\_writer.hpp:107

csv.hpp

stan::io::mcmc\_writer::write\_sample\_names

void write\_sample\_names(stan::mcmc::sample &sample, stan::mcmc::base\_mcmc \*sampler, M &model)

Outputs parameter string names.

**Definition:** mcmc\_writer.hpp:79

base\_mcmc.hpp

stan::mcmc::base\_mcmc::get\_sampler\_diagnostic\_names

virtual void get\_sampler\_diagnostic\_names(std::vector< std::string > &model\_names, std::vector< std::string > &names)

**Definition:** base\_mcmc.hpp:35

stan::mcmc::base\_mcmc::get\_sampler\_params

virtual void get\_sampler\_params(std::vector< double > &values)

**Definition:** base\_mcmc.hpp:31

stan::mcmc::sample::get\_sample\_params

void get\_sample\_params(std::vector< double > &values)

**Definition:** sample.hpp:55

stan::io::mcmc\_writer::write\_adapt\_finish

void write\_adapt\_finish(stan::mcmc::base\_mcmc \*sampler, Recorder &recorder)

Internal method.

**Definition:** mcmc\_writer.hpp:139

stan::mcmc::base\_mcmc::get\_sampler\_diagnostics

virtual void get\_sampler\_diagnostics(std::vector< double > &values)

**Definition:** base\_mcmc.hpp:38

stan::mcmc::sample::cont\_params

double cont\_params(int k) const

**Definition:** sample.hpp:30

stan::io::mcmc\_writer::write\_timing

void write\_timing(double warmDeltaT, double sampleDeltaT, Recorder &recorder)

Internal method.

**Definition:** mcmc\_writer.hpp:231

stan::mcmc::base\_mcmc::get\_sampler\_param\_names

virtual void get\_sampler\_param\_names(std::vector< std::string > &names)

**Definition:** base\_mcmc.hpp:29

stan::io::mcmc\_writer::write\_diagnostic\_names

void write\_diagnostic\_names(stan::mcmc::sample sample, stan::mcmc::base\_mcmc \*sampler, M &model)

Print diagnostic names.

**Definition:** mcmc\_writer.hpp:175

sample.hpp

stan::io::mcmc\_writer::write\_adapt\_finish

void write\_adapt\_finish(stan::mcmc::base\_mcmc \*sampler)

Prints additional info to the streams.

**Definition:** mcmc\_writer.hpp:157

stan::io::mcmc\_writer::write\_timing

void write\_timing(double warmDeltaT, double sampleDeltaT)

Print timing information to all streams.

**Definition:** mcmc\_writer.hpp:267

stan::io::mcmc\_writer

mcmc\_writer writes out headers and samples

**Definition:** mcmc\_writer.hpp:29

stan::mcmc::base\_mcmc::write\_sampler\_state

virtual void write\_sampler\_state(std::ostream \*o)

**Definition:** base\_mcmc.hpp:33

prob\_grad.hpp

stan::io::mcmc\_writer::write\_diagnostic\_params

void write\_diagnostic\_params(stan::mcmc::sample &sample, stan::mcmc::base\_mcmc \*sampler)

Print diagnostic params to the diagnostic stream.

**Definition:** mcmc\_writer.hpp:203

stan::mcmc::sample::get\_sample\_param\_names

void get\_sample\_param\_names(std::vector< std::string > &names)

**Definition:** sample.hpp:50

stan::mcmc::sample

**Definition:** sample.hpp:13

stan::common::sample

void sample(stan::mcmc::base\_mcmc \*sampler, int num\_warmup, int num\_samples, int num\_thin, int refresh, bool save, stan::io::mcmc\_writer< Model, SampleRecorder, DiagnosticRecorder, MessageRecorder > &writer, stan::mcmc::sample &init\_s, Model &model, RNG &base\_rng, const std::string &prefix, const std::string &suffix, std::ostream &o, StartTransitionCallback &callback)

**Definition:** sample.hpp:13

stan::mcmc::base\_mcmc

**Definition:** base\_mcmc.hpp:13


---

[ Stan Home Page ]

*© 2011–2014,
Stan Development Team.*
